# Minieditorial: O Polimorfismo Genético do Receptor Beta-Adrenérgico Tipo 1 Ser49Gly é Preditor de Morte em Pacientes Brasileiros com Insuficiência Cardíaca

**DOI:** 10.36660/abc.20200183

**Published:** 2020-05-12

**Authors:** Antonio Carlos Pereira-Barretto

**Affiliations:** 1 Departamento de Cardiopneumologia FMUSP São Paulo SP Brasil Departamento de Cardiopneumologia da FMUSP, São Paulo, SP – Brasil; 2 Instituto do Coração HCFMUSP São Paulo SP Brasil Instituto do Coração (InCor) do HCFMUSP - Serviço de Prevenção e Reabilitação,São Paulo, SP – Brasil; 3 Hospital Santa Marcelina São Paulo SP Brasil Hospital Santa Marcelina – Cardiologia, São Paulo, SP – Brasil

**Keywords:** Insuficiência Cardíaca/fisiopatologia, Insuficiência Cardíaca/mortalidade, Prognóstico, Polimorfismo Genético, Sistema Renina Angiotensina, Sistema Nervoso Simpático, Agonistas de Receptores Adrenérgicos beta-1, Agonistas de Receptores Adrenérgicos beta-2

A insuficiência cardíaca (IC) é uma doença que evolui com elevada morbi/mortalidade. No entanto, não são todos os pacientes que evoluem mal. Os sintomáticos e aqueles que necessitaram ser hospitalizados para tratamento são grupo de pior prognóstico. A intensidade dos sintomas vem se mostrando um bom preditor prognóstico. No entanto, em pacientes pouco sintomáticos temos capacidade muito mais limitada para identificar aqueles que terão pior evolução.^1^

No artigo “O Polimorfismo Genético do Receptor Beta-Adrenérgico tipo 1 Ser49Gly é Preditor de Morte em Pacientes Brasileiros com Insuficiência Cardíaca” nesta edição, os autores discutem um tema atual, no qual mostram que a evolução dos pacientes pelo menos em parte é relacionada ao seu perfil genético, e que este perfil determina a intensidade da IC e o desenvolvimento dos sintomas.^2^

Na IC a estimulação neuro-hormonal tem papel fisiopatológico importante, com os ensaios clínicos multicêntricos documentando de maneira cabal que o bloqueio dos sistemas renina angiotensina aldosterona e simpático hiperativados modificam a evolução da doença. E neste contexto o papel do sistema nervoso simpático está bem estabelecido e possivelmente tem o papel de maior vilão na história da IC. A resposta à estimulação neuro-hormonal não é igual em todos os pacientes e o polimorfismo genético influencia esta resposta.

A atividade simpática é mediada pelos receptores beta-adrenérgicos do tipo 1 e tipo 2.^3^ Tem-se avaliado o polimorfismo genético destes receptores e a atividade simpática difere conforme este polimorfismo. Para o receptor beta-1 dois polimorfismos têm sido mais estudados: o Ser19Gly e o Arg389Gly e para o receptor beta-2 também dois: Gly16Arg e Gln27Glu.^3^

O polimorfismo dos receptores beta-1 tem mostrado ter papel na incidência de IC, na resposta aos betabloqueadores, nos desfechos ecocardiográficos, na capacidade funcional, na incidência de arritmia cardíaca e na evolução clínica dos pacientes.^2 , 3^ No entanto, a maioria dos estudos foi realizado com população pequena e os resultados não tem mostrado resultados homogêneos, mas permitiu verificar que a constituição genética determina a evolução dos pacientes, inclusive a reposta ao tratamento.

Podemos verificar isto, em relação ao polimorfismo do receptor beta-2, quando no estudo FAST-Carvedilol. Na avaliação da sobrevida podemos documentar que analisando os pacientes considerando o polimorfismo, avaliando os genótipos DD-ID e II, que os portadores do polimorfismo II tiveram maior mortalidade do que as variantes DD e ID. Mas, o resultado mais interessante foi que estes pacientes II, quando receberam dose de carvedilol otimizada (>50% da dose alvo), apresentaram expressiva redução de mortalidade, enquanto nos portadores das variantes DD e ID a dose não modificou a evolução.^4^ Como resultado do estudo observamos que o grupo II tratado com dose baixa de carvedilol apresentou chance 6 vezes maior de morrer do que o grupo que recebeu dose otimizada de carvedilol.^4^

Na mesma linha de pesquisa, no estudo MERIT-HF, analisando polimorfismo dos receptores Beta-1 observou-se que havia pacientes recebendo doses elevadas de betabloqueador que não respondiam ao tratamento, ao lado de outros que apresentavam melhora importante.^3^ No estudo BEST o polimorfismo genético foi imputado na falta de resposta ao betabloqueador bucindolol. Este foi um dos poucos estudos multicêntricos com número expressivo de pacientes que analisou prospectivamente o papel do polimorfismo na resposta terapêutica de um betabloqueador. O polimorfismo foi analisado neste ensaio multicêntrico com mais de 1.000 pacientes e mostrou que os pacientes com o polimorfismo selvagem Gly389 do receptor beta-1 não responderam ao tratamento com bucindolol. Por outro lado, aqueles sem este polimorfismo tiveram redução de mortalidade com o bucindolol.^3^ Os pesquisadores consideram que os dados sobre o polimorfismo não são sempre concordantes e que no momento é melhor não utilizar esta ferramenta para orientar o tratamento.^2^

Mas, seu papel na evolução dos portadores de IC continua nos enriquecendo de informações permitindo um melhor entendimento desta complexa síndrome. Os estudos vêm mostrando que a resposta do sistema adrenérgico mediada pelas variantes genéticas dos adrenorreceptores centrais ou periféricos tem papel na fisiologia da IC. Como já apresentado essa variabilidade interindividual modifica inclusive o prognóstico da IC, com alguns pacientes apresentando mais eventos cardíacos a despeito da estabilidade clínica moderada disfunção ventricular e preservada capacidade de exercício. Inversamente, outros, classificados clinicamente como portadores de IC avançada, evoluem com uma prolongada e não esperada sobrevida. E que os dados mostraram que parte das diferenças percebidas na eficácia dos betabloqueadores, bem como a variabilidade de respostas a estes, podem ser atribuídas a algumas variações genéticas que afetam os receptores beta e suas vias de sinalização.^2 , 3^

No Brasil o polimorfismo dos receptores beta-1 foi objeto de estudo no Rio de Janeiro e no Rio Grande do Sul.^2 , 5^

No estudo objeto deste minieditorial, os autores destacam que o receptor cardíaco beta-adrenérgico do tipo 1 (Rβ1) é a principal estrutura responsável por mediar os efeitos da adrenalina e que a estimulação sustentada desse sistema promove múltiplos efeitos deletérios, destacando-se a cardiotoxicidade. As variantes genéticas estão associadas a diferente atividade desse receptor. Os autores estudaram o polimorfismo genético identificado na posição 145 do nucleotídeo, na qual a serina é substituída pela glicina na posição 49 (Rβ1-Ser49Gly).^2^

Este trabalho descreve, em população brasileira, a relação entre os genótipos do polimorfismo genético do receptor beta1 – Ser49Gly e a evolução clínica em 178 pacientes com IC, com seguimento médio de 6,7 anos.^2^ Trata-se de trabalho com genotipagem do Ser49Gly no contexto da IC com maior tempo de seguimento já publicado. Seu principal achado foi a associação do polimorfismo genético Gly-Gly com um efeito protetor para desfechos clínicos, com melhor evolução clínica avaliada pela classe funcional NYHA e menor risco de morte. O maior tempo de seguimento permitiu avaliar melhor os aspectos evolutivos da IC e verificar que alelo Gly está associado a melhor evolução clínica; no entanto, observou-se uma potencial influência da etnia sobre esses genótipos, invertendo esse comportamento benigno em algumas populações. Ponto importante foi permitir avaliar prognóstico em pacientes pouco sintomáticos, ampliando a acuidade da avaliação prognóstica também para esses pacientes.

Quanto ao prognóstico os resultados deste estudo foram semelhantes aos obtidos no trabalho do Rio Grande do Sul,^5^ agregando importante contribuição ao identificar que pacientes com o perfil Gly-Gly, menos frequente, permaneceram pouco sintomáticos durante todo o seguimento, identificando desta forma grupo de pacientes com menor potencial evolutivo.^3^

No entanto, deve-se ressaltar que a amostra testada é pequena e estudos confirmatórios são necessários para a verificação dessa hipótese, no intuito de mostrar se as variantes genéticas dos receptores beta-adrenérgicos podem ajudar a identificar os pacientes com IC que irão ter menor progressão da doença e se serão mais responsivos aos betabloqueadores e, como consequência, uma melhor evolução clínica.

Os resultados permitem supor que, no futuro, antes de iniciarmos um tratamento com bloqueador neuro-hormonal ou betabloqueador, será indicado identificar o perfil genético e prescrever os medicamentos somente para os responsivos.
