# Supplementary material for: Minieditorial: O Polimorfismo Genético do Receptor Beta-Adrenérgico Tipo 1 Ser49Gly é Preditor de Morte em Pacientes Brasileiros com Insuficiência Cardíaca
Source: Arq Bras Cardiol. 2020 May 12;114(4):625–6. [Article in Portuguese] doi: 10.36660/abc.20200183 (PMC9744336; doi:10.36660/abc.20200183)
Supplement: Translation

# Translation: Short Editorial: Ser49Gly Beta1-adrenergic Receptor Genetic Polymorphism as a Death Predictor in Brazilian Patients with Heart Failure

**Authors:** Antonio Carlos Pereira-Barretto

**Affiliations:** 1 Brazil Departamento de Cardiopneumologia da FMUSP, São Paulo, SP – Brazil; 2 Brazil Instituto do Coração (InCor) do HCFMUSP - Serviço de Prevenção e Reabilitação, São Paulo, SP – Brazil; 3 Brazil Hospital Santa Marcelina – Cardiologia, São Paulo, SP – Brazil

**Keywords:** Heart Failure/physiopathology, Heart Failure/mortality, Prognosis, Genetic,Polymorphism, Sympathetic Nervous System, Adrenergic beta-1, Receptor Agonists, Adrenergic beta-2

Heart failure (HF) is a disease that develops into high morbidity/mortality. However, not all patients have a bad evolution. Symptomatic patients and those who require hospitalization for treatment comprise the group with the worst prognosis. Symptom intensity has shown to be a good predictor of prognosis. However, in less symptomatic patients, we have a much more limited capacity to identify those who will have a worse evolution.^1^

In the article “Ser49Gly Beta1-adrenergic Receptor Genetic Polymorphism as a Death Predictor in Brazilian Patients with Heart Failure”, published in this issue, the authors discuss a current topic, in which they show that patient evolution is, at least in part, related to their genetic profile, and that this profile determines the intensity of HF and symptom development.^2^

Neurohormonal stimulation has an important pathophysiological role in HF, and multicentric clinical trials have fully documented that the blockade of the overactivated renin-angiotensin-aldosterone and sympathetic systems modifies the disease evolution. And in this context, the role of the sympathetic nervous system is well established and possibly has the role of the greatest villain in the history of HF. The response to neurohormonal stimulation is not the same in all patients and the genetic polymorphism influences this response.

The sympathetic activity is mediated by type 1 and type 2 beta-adrenergic receptors.^3^ The genetic polymorphism of these receptors has been evaluated and the sympathetic activity differs according to the polymorphism. For the beta-1 receptor, two polymorphisms have been more frequently studied: Ser19Gly and Arg389Gly and for the beta-2receptor, also two polymorphisms: Gly16Arg and Gln27Glu.^3^

The beta-1 receptor polymorphism has been shown to play a role in the incidence of HF, response to beta-blockers, echocardiographic outcomes, functional capacity, incidence of cardiac arrhythmia and the clinical evolution of patients.^2 , 3^ However, most studies were carried out with small populations and the outcomes have not shown homogeneous results, although it has been possible to verify that the genetic constitution determines patient evolution, including treatment response.

One can verify this fact, in relation to the beta-2 receptor polymorphism, in the FAST-Carvedilol study. When assessing survival, one can document that by analyzing patients considering the polymorphism, evaluating the DD-ID and II genotypes, the carriers of the polymorphism II had higher mortality than the DD and ID variants. However, the most interesting result was that patients II, when they received an optimized dose of carvedilol (> 50% of the target dose), showed a significant reduction in mortality, while in patients with the DD and ID variants, the dose did not change the evolution.^4^ As the study result, we observed that group II treated with a low dose of carvedilol had a 6-fold greater chance of dying than the group that received an optimized dose of carvedilol.^4^

In the same line of research, in the MERIT-HF study, when analyzing the Beta-1 receptor polymorphism, it was observed that there were patients receiving high doses of beta-blockers who did not respond to treatment, whereas others showed significant improvement.^3^ In the BEST study, the genetic polymorphism was associated to the lack of response to the beta-blocker bucindolol. This was one of the few multicenter studies with a significant number of patients that prospectively analyzed the role of the polymorphism in the therapeutic response to a beta-blocker. The polymorphism was analyzed in this multicenter trial with more than 1,000 patients and showed that patients with the wild beta-1 receptor Gly389 polymorphism did not respond to treatment with bucindolol. On the other hand, those without this polymorphism had reduced mortality with bucindolol.^3^ The researchers consider that the data on the polymorphism are not always consistent and that at the moment it is better not to use this tool to guide treatment.^2^

However, its role in the evolution of HF patients continues to supply us with information, allowing a better understanding of this complex syndrome. The studies have shown that the adrenergic system response mediated by the genetic variants of central or peripheral adrenoceptors has a role in the physiology of HF. As already shown, this inter-individual variability even changes the prognosis of HF, with some patients showing more cardiac events despite the moderate clinical stability of ventricular dysfunction and preserved exercise capacity. Conversely, others, clinically classified as having advanced HF, evolve with a prolonged and unexpected survival. Moreover, the data showed that part of the perceived differences in the effectiveness of beta-blockers, as well as the variability of responses to the latter can be attributed to some genetic variations that affect beta receptors and their signaling pathways.^2 , 3^

In Brazil, the beta-1 receptor polymorphism has been studied in Rio de Janeiro and Rio Grande do Sul.^2 , 5^

In the study discussed in this short editorial, the authors emphasize that the cardiac beta-1 adrenergic receptor (Rβ1) is the main structure responsible for mediating the effects of adrenaline and that the sustained stimulation of this system results in multiple deleterious effects, especially cardiotoxicity. Genetic variants are associated with different activities of this receptor. The authors studied the genetic polymorphism identified at position 145 of the nucleotide, in which serine is replaced by glycine at position 49 (Rβ1-Ser49Gly).^2^

This study describes, in a Brazilian population, the association between the genotypes of the Ser49Gly Beta1-adrenergic Receptor Genetic Polymorphism and the clinical evolution in 178 patients with HF, with a mean follow-up of 6.7 years.^2^ This is a study with Ser49Gly genotyping in the context of HF with the longest follow-up time ever published. Its main finding was the association of the Gly-Gly genetic polymorphism with a protective effect for clinical outcomes, with better clinical evolution assessed by NYHA functional class and lower risk of death. The longer follow-up allowed us to better assess the HF evolution aspects and to verify that the Gly allele is associated with better clinical evolution; however, there was a potential influence of ethnicity on these genotypes, reversing this benign behavior in some populations. An important point was to allow the assessment of prognosis in little symptomatic patients, increasing the accuracy of the prognostic evaluation for these patients, as well.

As for the prognosis, the results of this study were similar to those obtained in the study carried out in Rio Grande do Sul,^5^ adding an important contribution by identifying that patients with the Gly-Gly profile, which is less frequent, remained little symptomatic throughout the follow-up, thus identifying a group of patients with lower evolution potential.^3^

However, it should be noted that the assessed sample is small and confirmatory studies are necessary to verify this hypothesis, aiming to show whether the genetic variants of beta-adrenergic receptors can help to identify patients with HF who will have a lower disease progression and whether they will be more responsive to beta-blockers and, as a consequence, have a better clinical evolution.

The results allow us to suppose that, in the future, before starting a treatment with neurohormonal blockers or beta-blockers, the genetic profile will be identified, and medications will be prescribed only to those who are responsive to them.
